# MCR: Open-Source Software to Automate Compilation of Health Study Report-Back

**DOI:** 10.3390/ijerph18116104

**Published:** 2021-06-05

**Authors:** Erin Polka, Ellen Childs, Alexa Friedman, Kathryn S. Tomsho, Birgit Claus Henn, Madeleine K. Scammell, Chad W. Milando

**Affiliations:** 1 Department of Environmental Health, Boston University School of Public Health, 715 Albany St., Boston, MA 02118, USA; polkaen@bu.edu (E.P.); lexf@bu.edu (A.F.); bclaus@bu.edu (B.C.H.); mls@bu.edu (M.K.S.); 2 Abt Associates, Division of Health and the Environment, 6130 Executive Blvd, Rockville, MD 20852, USA; ellen.childs@gmail.com; 3 Department of Health Policy and Law, Boston University School of Public Health, 715 Albany St., Boston, MA 02118, USA; 4 Department of Environmental Health, Harvard T.H. Chan School of Public Health, 677 Huntington Ave., Boston, MA 02115, USA; tomshok@g.harvard.edu

**Keywords:** report-back, health equity, community engagement, data sharing, software

## Abstract

Sharing individualized results with health study participants, a practice we and others refer to as “report-back,” ensures participant access to exposure and health information and may promote health equity. However, the practice of report-back and the content shared is often limited by the time-intensive process of personalizing reports. Software tools that automate creation of individualized reports have been built for specific studies, but are largely not open-source or broadly modifiable. We created an open-source and generalizable tool, called the Macro for the Compilation of Report-backs (MCR), to automate compilation of health study reports. We piloted MCR in two environmental exposure studies in Massachusetts, USA, and interviewed research team members (*n* = 7) about the impact of MCR on the report-back process. Researchers using MCR created more detailed reports than during manual report-back, including more individualized numerical, text, and graphical results. Using MCR, researchers saved time producing draft and final reports. Researchers also reported feeling more creative in the design process and more confident in report-back quality control. While MCR does not expedite the entire report-back process, we hope that this open-source tool reduces the barriers to personalizing health study reports, promotes more equitable access to individualized data, and advances self-determination among participants.

## 1. Introduction

In recent decades, the health science community has increasingly shared individualized results with participants of research studies, a practice we and others refer to as “report-back.” Reporting back health data has historically been limited by researcher (and institutional review board) concern over sharing results, especially results lacking relevant reference values or clear pathways to clinical remediation, e.g., environmental exposures to unregulated chemicals [1]. Compiling reports of individualized results can also be extremely time-intensive. Owing to these barriers, most post-study reports are limited to aggregated results or clinical findings with known health significance. Scientists (and activists) have made ethical arguments for a change to this status quo [2]. Participants are essential in health research, and sharing results is one way of “demonstrating respect and gratitude for their contributions” [1]. Individualized results can improve participant comprehension of health risks [3], guide participant self-determination in health-related decisions [4], and are valued by participants even if the implications of the results are unclear or uncertain [4,5,6,7,8]. In addition, the unequal distribution of adverse health impacts in vulnerable communities has further elevated the need to share and contextualize individualized results; greater access to knowledge is a key step in promoting justice and addressing health inequities [4,8]. Study participants were more likely to seek information or take action to reduce exposures after receiving reports personalized with their regional and demographic characteristics or personal exposures as compared to actions taken following receipt of reports containing only generalized results [9,10,11]. Efforts to share study results via report-back and to evaluate the report-back process as well as feedback from community groups and study participants have led to recent US federal guidance documents encouraging increased sharing of individualized results in health studies [1].

However, many barriers remain in reporting individual results from research studies. Much of the focus of report-back literature is in communicating results of studies with clear clinical significance [1], but guidance on communicating environmental exposures with undetermined clinical significance is less common [7,12]. New methods of measuring exposure have outpaced ethical and legal precedent for reporting [4,5]. Communicating results becomes increasingly time-intensive in proportion to a study’s complexity [1]. Financial constraints, lack of institutional support, and lack of researcher expertise in reporting can further limit the report-back process [2]. Personalizing results, communicating uncertainty, and compiling high-level and individual results takes careful consideration and staff resources, the logistical burden of which likely causes many studies to limit the degree of personalization in report-back [1,8,13]. Researchers also recognize that manual compilation of reports increases the chances of human error [1,8]. However, failure to report group-level or individual results to participants further perpetuates, rather than mitigates, existing disparities in health and exposures [1].

To overcome some of these barriers, researchers have used or created software tools that aid in preparing individualized reports. Repositories of visualizations that improve health communication are a helpful first-order solution [14,15,16,17]. Online tools allow researchers to input cleaned datasets (e.g., participant biomonitoring data), prepare individual summaries and graphs, and observe participant interaction with their individual data [18,19]. More resource-intensive are project-specific digital tools that use community-based participatory methods to generate infographics or templates for individual reports [9,10,11,20,21,22]. However, most available tools are limited to specific datasets, offer limited or non-customizable visualization options, or are not open-source and thus not available for widespread use.

We present a generalizable open-source tool called the Macro for the Compilation of Report-backs, or MCR, which provides researchers in health studies with a flexible and scalable method for compiling individual reports. MCR aids researchers by populating a report template with individual numerical, text, and graphical results for each study participant. We created MCR and piloted it in two environmental exposure studies in Massachusetts, USA. We then interviewed research team members from each study (*n* = 7) to compare the report-back process before and after the implementation of MCR.

## 2. Materials and Methods

### 2.1. MCR Description

MCR is a macro-enabled Microsoft Excel workbook that enables users to automatically compile detailed and individualized reports for study participants. MCR source code, example files, and a detailed instruction manual are available in an online repository at https://github.com/cmilando/reportback-vba (available as of 1 June 2021). The Visual Basic for Applications (VBA) functions that compose MCR sequentially insert a participant’s numerical, text, and graphical results into a report-back template (a Microsoft PowerPoint file) and save individual reports (as either a PowerPoint file or a PDF file). Users of MCR create report-back templates, prepare individualized participant results, run MCR, and then distribute reports to participants. We summarize the components and implementation of MCR below and provide an example of a blank template and an “individualized” report in the Supplementary Materials (note that the supplemental report-back template demonstrates MCR’s capabilities rather than provides an example of the best report-back practices).

**Figure 1:**
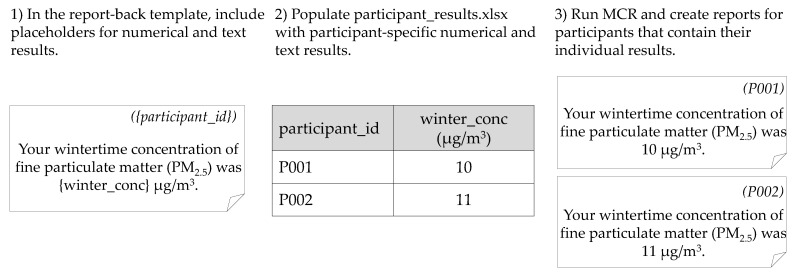
Visual representation of the steps necessary to insert numerical and text data into the report-back template using MCR.

**Figure 2:**
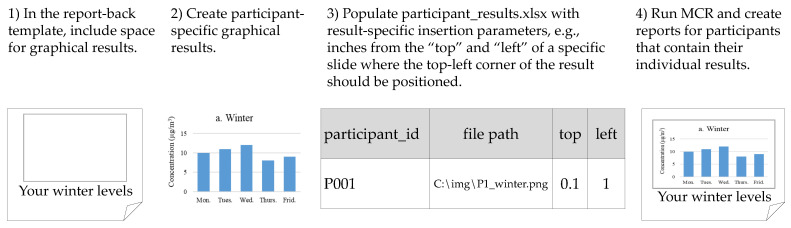
Visual representation of the steps necessary to insert graphical results into the report-back template using MCR.

The report template contains the layout for each page of the individual report. Template design is at the discretion of the research team. Generally, templates contain generic information present in all reports (e.g., a description of common indoor pollution sources), blank space for graphical results, and placeholders for individualized numerical or text results. Placeholders are composed of (variable) bracketing characters (e.g., “{“and“}”) and a unique identifier for the result to be inserted (e.g., “{participant_name}”). The inserted text matches the format of each specific placeholder. Researchers prepare individual numerical, text, and graphical results using software of their choice; the research teams in the applications below used R [23] and VBA. A Microsoft Excel workbook (e.g., participant_results.xlsx) is the container for all participant results, with worksheets for participant-specific numerical and text results (e.g., words, phrases, sentences), graphic results, and instructions for any special formatting characters (e.g., subscripts in chemical formulae). For numerical and text results, each worksheet row corresponds to results for an individual participant, with column headers corresponding to specific template placeholders and column cells representing individualized results to be inserted into the report-back template. Figure 1 provides an example of insertion of numerical results into an individual report and Figure 2 provides an example of insertion of graphical results. For the sheet of graphical results, each row corresponds to a single graphical result for a specific participant and contains the graphical result file path, the report-back page number, and the insertion parameters (e.g., changes to the image aspect ratio, distance from the top or side of the specified page).

### 2.2. MCR Applications

In early 2020, we piloted MCR in two environmental exposure studies in Massachusetts, USA, as part of the Center for Research on Environmental and Social Stressors in Housing Across the Life Course (CRESSH) and the Assessing Children’s Environmental Exposures (ACHIEVE) project. Each study team had recently completed at least one report-back using documents created manually for each participant. In each study, we used MCR to complete a subsequent round of report-back. Following implementation of MCR, we interviewed team members from both studies and asked them to describe the report-back process with and without the use of MCR. We then performed qualitative analyses to identify key benefits and challenges of MCR implementation.

#### 2.2.1. CRESSH

The Center for Research on Environmental and Social Stressors in Housing Across the Life Course (CRESSH) is an environmental health disparities (EHD) research center funded by the US Environmental Protection Agency and the National Institute on Minority Health and Health Disparities involving researchers at Boston University School of Public Health and Harvard T.H. Chan School of Public Health. The CRESSH project that used MCR was the Home Observation and Monitoring Exposure (HOME) study, which involved collection of indoor air quality data from 150 homes in two environmental justice communities in the Greater Boston area: 72 homes in the city of Chelsea and 74 homes in the Boston neighborhood of Dorchester [24,25]. Report-back for HOME consisted of general and individualized information which characterized common routes of exposure to indoor air contaminants, as well as numerical, text, and graphical representations of participants’ in-home air concentrations of these contaminants. The study team completed the report-back for Chelsea without MCR in summer 2018, manually creating individualized reports for each participant [26,27]. For the report-back in Dorchester (winter 2020), the study team used MCR to generate reports using numerical, text, and graphical results prepared in R and Microsoft Excel.

#### 2.2.2. ACHIEVE

Assessing Children’s Environmental Exposures (ACHIEVE) is a community-initiated pilot study conducted by researchers at Boston University School of Public Health and by community members in Holliston, MA, USA, to assess early life exposure to manganese in their local drinking water. Researchers worked alongside community members to collect residential tap water and previously shed baby teeth, a biomarker of retrospective exposure, and then reported estimates of the children’s previous (teeth) and current (water) manganese exposure [28]. In the prior report-back, researchers created letters that provided individual-level information on tap water manganese levels for 19 homes in winter 2019 and group-level information on tooth manganese levels for 28 participants in fall 2019. Subsequently, in winter 2020, researchers used MCR and Microsoft Excel to complete the third round of report-back for individual-level information on manganese in repeated tap water samples for 21 homes.

#### 2.2.3. Qualitative Analysis

One member of our team (E.C.) conducted qualitative interviews over the phone with seven researchers from the CRESSH and ACHIEVE study teams. Interviewees included principal investigators (*n* = 2), doctoral students (*n* = 2), project coordinators (*n* = 2), and a project manager (*n* = 1). Boston University Medical Campus Institutional Review Board approved our study methods and interview questionnaire (approval number H-40282, interview questions listed in Table 1). E.C. removed any identifying information from the interview transcripts, and then three members of our research team (E.C., E.P., C.M.) coded de-identified interview transcripts using a directed content analysis process [29] in NVivo (QSR International Pty Ltd., Doncaster, Australia, version 12, 2019). Codes highlighted the benefits and challenges of the manual report-back process and the report-back process using MCR.

## 3. Results

### 3.1. Report-Back Using MCR

We conducted interviews by phone during July and August 2020, and each interview lasted approximately 20 min. Table 2 summarizes characteristics of the report-back output for both manual and MCR approaches for the CRESSH HOME and ACHIEVE projects. Following MCR implementation, reports included more forms of individualized results: graphical, numerical, and text results, summaries and interpretations of results, and engagement questions. Using coded algorithms in R, the CRESSH team members created personalized interpretations of each graphic, e.g., “Your home had high concentrations of fine particulate matter (PM_2.5_) in the wintertime,” and used MCR to insert this text below each graphical result. In addition, researchers created engagement questions specific to each participant’s results, e.g., “What are some ways you could reduce fine particulate matter (PM_2.5_) indoors during the winter months?” For ACHIEVE, the report-back letters also became more complex, including individualized graphic and expanded numerical results. In both studies, the time to compile results was much shorter using MCR, requiring only minutes versus hours of manually cutting and pasting results.

### 3.2. Benefits and Challenges

Through the qualitative data analysis, we identified benefits and challenges of manual report-back and report-back with MCR. We describe those in turn below.

#### 3.2.1. Benefits and Challenges of Manual Report-Back

*Benefits*. When researchers created reports manually, they became familiar with each participant’s results. A working knowledge of the results of all participants was helpful in the report-back design process; one researcher noted “the fact that I had to look through these different individuals’ figures so many times, … inform[ed] some of our discussion on how figures should be created for interpretability and human reaction, motivational reaction and things like that.” A researcher also pointed out that this in-depth understanding was useful in community interactions, saying, “having sat with each person’s data for a long time and knowing their specific graphs really intimately helped me [respond] more quickly when we were having conversations in those workshops with participants.” In manual report-back compilation, study team members chose a simplified report-back design that favored ease of creation and participant comprehension. These constraints necessitated many iterations of intra- and inter-team review of report-back templates, which, as a researcher described, had positive impacts on the report-back process: “the whole process really benefited from the number of eyes that saw the templates, figures and tables.”

*Challenges*. Individualizing reports by hand is time-intensive. Researchers described the manual report-back process as being “very inefficient” and “frustrating knowing that there was obviously a better way to automate it.” As one team member commented, “anything is better than having to do it one at a time.” Another reflected, “It was super time consuming for her [researcher compiling reports], I think it was really stressful. I think she worked non-stop when she was doing all of those.” The time-intensive nature of the work had downstream effects, requiring researchers to choose a final report design for all participants prior to production of reports, “We were [trying to] make decisions, but not seeing the results until everything was done.”

Researchers reported that the amount of time needed to compile each report manually limited individualization and creativity in the report-back design. As one team member noted, “You can only make them so individualized when you’re doing them by hand.” Researchers found they faced “a trade-off with creating these things and making them really useful and accessible and also trying to get them back to the participants in a time frame that’s reasonable.” They finalized layouts prior to compilation, which favored fewer simpler graphics. According to one researcher, “You wouldn’t spend as much time getting creative and consulting as you maybe would if it didn’t take as much time to individually craft all of them.”

Inserting individualized data by hand also presented challenges of data accuracy. “There’s a lot of room for error in the transcription of data from one source to another,” noted one researcher. Double-checking the reports for accuracy was another researcher’s “biggest worry,” as they were concerned they were “going to give somebody incorrect data because of human error.” Multiple team members helped spot-check manually created reports, and one researcher noted having to go back to check each report “2–3 times, which takes a long time obviously for 72 participants when you’re checking individual data points.”

#### 3.2.2. Benefits and Challenges of Report-Back with MCR

*Benefits*. The chief benefit of using MCR in report-back was the reallocation of researcher time from manual entry of results to report-back design. The ability to rapidly generate draft reports for all participants within minutes allowed team members to view a “variety of options” and choose which one “makes the most sense for what we’re trying to communicate.” This greatly expanded the iterative design process; according to one researcher,

“[Team member] has been able to, like within a week, turn around a variety of requests to create alternate graphics and visuals… she can do it so quickly and present us a variety of options to go back over and see what one makes the most sense for what we’re trying to communicate. We definitely couldn’t have played around that much with it in that first report-back.”

As described above, final reports in both studies contained an increased number of individualized results with greater complexity, and team members reported feeling less pressured by time constraints to choose between reporting aggregated or individual results.

With MCR, researchers reported the ability to be more creative in reports and include individualized output to facilitate improved decision-making for participants. As one team member explained, the process of report-back “has changed in that we’ve been able to think of new and creative ways to report-back this data.” According to another,

“We’ve been able to apply some tools from the health literacy field again to reduce the level of numeracy and graphicacy that’s required to engage in the material. And it’s been helpful to do that iterative process of ‘here’s the graph that we have, how can we reduce the [graphicacy] demand [for the reader]?’”

Another reported, “We’ve thought more thoroughly about the data and what the message will be because we’ve had more time to do that, instead of knowing that we’re going to individually craft letters.” Speaking about MCR and figures, one researcher replied that the “tool allows us to make that graphic more advanced or more complex to handle the complexity of the data now.” Another reflected, “within the report-back there’s room for a lot more combinations of results and programming how they’re displayed, which is pretty cool.”

Researchers also noted that using MCR increased consistency in messaging across all reports created for a community. Referring to individualized text, one team member said, “I think applying [MCR] makes it very systematic… it’s not on us to come up with an interpretation, we might misinterpret something... when that’s not appropriate. It creates a structure that’s I guess more reliable.” A driver of this increased consistency was the increased capacity to find outliers in data or graphs. As put by one team member,

“Now we can look at all the data and say ‘oh this isn’t going to look right because these people are too low or too high so we have to make some changes to adopt and better understand how they will look.’ We can see everything ahead of time, so we can pre-plan how we’re going to try to help get people to understand their results.”

Using MCR increased confidence in report-back quality control. According to one research team member, “there’s less human error in this automated process, so that makes me feel better about sending these out.” Another reported, “the work has changed in that there are less errors in theory because I’m not handcrafting [reports].” Even when errors or typos were found, researchers still found that MCR was helpful; according to one researcher, “It saves time at the end of compiling everything. If you need to make a change to the underlying template or the graph, you just make it to the baseline code or the template, and that can change it to everybody, immediately.”

Researchers also reported being excited about the expanded use of MCR in a range of research settings. One team member noted the benefit for studies with limited resources for community engagement, “[I] think having a tool that significantly cuts back the time it takes to do it, it kind of ensures, there’s quality assurance built in, I feel like it reduces that barrier for other research groups who may see it as less of an expense to build in a report-back process into the research plan.” Once researchers make a template, it can be reused and reproduced for multiple study rounds, benefiting long-term studies that plan to produce multiple reports. Another researcher remarked that,

“Once you get into bigger studies, like maybe something that’s a cohort of a thousand, the ability to create those individual reports by hand just disappears, it’s not feasible time-wise, and I think there’s a growth and interest in the environmental health field and community engagement in particular to provide this data back to people. I think it’s going to be useful as larger and larger studies are providing that data back to people. I think once you start to get into a couple hundred or a thousand, you don’t have a choice but to automate it.”

MCR was noted as providing a study the ability to create “the infrastructure, or platform, to be able to scale up eventually.”

*Challenges*. Using MCR presented minor challenges to researchers in the time required to create the template and the master file and in the technical expertise needed to troubleshoot MCR output. Although coding knowledge is not required to run MCR, first-time users must make a commitment to learn how to use the workbook, i.e., create a template in Microsoft PowerPoint, prepare participant results, and troubleshoot inaccuracies in draft reports (e.g., correct image placement). Additionally, generating complex graphics took time (e.g., in R or Excel, rather than with MCR). One team member mentioned “there’s a bit of time up front that’s needed to craft and develop the right graphic tool, so there’s a bit of ramp up time in refining what that looks like.” Complex reports with more individualized output required more front-end time dedicated to coding logic algorithms, and MCR did not eliminate the need for spot-checking reports. The version of MCR used in these studies functioned primarily with Microsoft Office products (Excel and PowerPoint), which some users noted may be limiting to other research teams. Finally, researchers agreed the increased front-end time necessary for MCR may not always be justified given reports that are simpler in design or studies with smaller numbers of participants.

Table 3 summarizes qualities of the report-back process and whether the manual report-back and MCR process fit the identified qualities.

## 4. Discussion

MCR provides multiple benefits to researchers engaged in health sciences. MCR facilitates the implementation of effective communication strategies, such as the coupled insertion of individual and summarized results in text and graphical formats [30], an advantage over the standard process of providing participants with generic text summaries based on “flagged” data [4]. The opportunity for increased individualization can help account for participant variation and improve engagement with historically underserved populations. Importantly, automating the individualization and compilation process provides the infrastructure to scale up a project’s reports for large cohorts even with limited staff resources or complex results. With the potential to rapidly create multiple report-back drafts for any cohort size, researchers can shift their time to focus on reducing numeracy and graphicacy demands or on including multiple representations of results and participant-specific explanations of individualized results important for communication of health data [1,12,31,32]. The ability to easily preview report-back drafts can inspire data exploration and help researchers to plan, understand, and communicate participant results [19]. Finally, MCR enables improved reporting accuracy and greater consistency across all reports for both aggregated and individualized data, which was noted to increase researcher confidence in reports and can provide opportunities for easier evaluation of effectiveness in communication of results [12].

Compared to other tools and programs designed to improve the report-back process and communication of health results [9,10,11,15,16,17,18,19,20,21], MCR is unique in that it allows flexible creation of any report-back design desired by researchers, and the open-source design makes it accessible to any research or community team. While MCR does not expedite certain elements of the report-back process (e.g., the time required to compile data, design materials and templates, and meet with community members for feedback), the design process is not independent of the compilation process. Knowing that reports can be compiled efficiently can influence the decision to construct more personalized templates and make it easier to respond to feedback from participants and community partners during the report-back design process. Overall, MCR provides researchers with the potential to create more detailed reports without the manual effort usually required for report compilation.

Using MCR to compile reports in public health studies has multiple downstream benefits for participants. The time and effort saved during the compilation process allows greater opportunity for community-based participatory research approaches that increase engagement and inclusion among participants [1,7,11,33], as well as increased focus on implementing health literacy strategies that improve the accessibility and decrease the complexity of report-back materials [34]. Increasing accessibility of environmental health materials can empower study participants by increasing their access to critical health equity data [1,3,4,7,35]. Personalized messaging can better guide participants in understanding their data, motivate action-taking behaviors, and present appropriate suggestions, especially for persons with higher exposures [1,9,10,35]. Users of MCR should always adhere to the recommended reporting practices and prioritize participant needs and desires in the report-back design as opposed to inclusion of a large quantity of individualized results without clear interpretations.

The MCR version used in these studies was in development throughout its implementation. Researchers were limited to using MCR in a specific environment (Windows) with specific Microsoft Office products (Excel and PowerPoint) and with a simple graphical user interface. Several researchers from both teams cited minor technological barriers in working with MCR for the first time; many of these barriers were addressed in updates to MCR source code during implementation. Increased individualization was in some cases accompanied by a more complicated results creation process, often requiring additional coding skillsets (however, these were employed prior to use of MCR). Although similar expedited report-back creation could be accomplished using document-scripting software (e.g., LaTEX), such a method of report-back creation may exceed the technological capacity of many research teams and furthermore may not be as flexible as MCR, e.g., in manual repositioning of report-back elements in a template. Interviewing more researchers and teams who use MCR as well as participants who both engage with report-back design and receive report-backs created using MCR could uncover a greater set of benefits and challenges in report-back creation that future MCR versions would address. Despite some learning curves, the overall benefits to both projects outweighed the challenges, as both teams reported that learning to use MCR proved advantageous over compiling reports manually.

## 5. Conclusions

We developed and implemented MCR, a generalizable tool for compiling individualized reports for participants in exposure or health studies. In two studies that used MCR, researchers designed reports that contained more creative and individualized report components, e.g., individually personalized engagement questions. The automated compilation process was efficient, reduced the potential for human error, and increased researcher confidence in report-back quality control. The technical skillset required to use MCR is basic proficiency with Microsoft Excel and PowerPoint, programs used across public health disciplines. While MCR does not expedite the entire report-back process, we hope that it provides researchers and community advocates with an expanded ability to be creative in their report-back processes, promotes more equitable access to individualized data, and advances self-determination and health equity among research participants.

## Figures and Tables

**Table 1:** Semi-structured interview questions for the research team members using MCR in a subsequent report-back.

1. Tell me a bit about the previous process for preparing environmental or health data in individual reports for research participants (i.e., “report-back”).
a. What were the considerations for the methods of data presentation (e.g., in text, tables, or figures) b. How much time do you think each report took? c. What was the process for review and editing by the research team?
2. What were some of the benefits of the way these reports were developed before?
3. What were some of the limitations of this report development process?
4. With MCR, what has changed about your work?
a. How much time does each report take? b. How has this changed the considerations for the methods of data presentation (e.g., in text, tables, or figures)? c. How has this changed the scope or goals for future environmental health projects?
5. What are the benefits of MCR?
6. What are the limitations of MCR?
a. How much technical expertise is needed to adapt or modify MCR to develop new reports?
7. What suggestions do you have to adapt or modify MCR?

**Table 2:** Characteristics of reports created manually and using MCR.

Study	Report Component	Manual Report-Back	Report-Back with MCR
CRESSH HOME	Number of pages	7	19
	Number of individualized numerical or text results	4	23
	Number of individualized tables	2	6
	Number of individualized graphs	4	8
	Other	ten generic engagement questions	six individualized engagement questions
ACHIEVE ^1^	Number of pages	2	2
	Number of individualized numerical or text results	0	5
	Number of individualized tables	0	1
	Number of individualized graphs	0	1

^1^ In ACHIEVE, manual report-back was for reporting manganese levels in previously shed baby teeth, whereas report-back with MCR was for reporting manganese levels in residential tap water.

**Table 3:** Report-back process qualities for manual reporting and reporting with MCR.

Report-Back Process Qualities	Manual Report-Back	Report-Back with MCR
▪ Time spent on manual entry and report compilation/review	✓	
▪ Time spent on report design	✓	✓
▪ Confidence in report data quality; low potential for data entry errors		✓
▪ Individualized results	✓	✓
▪ Technical expertise (Excel, PowerPoint) required	✓	✓
▪ Adaptable for large-scale studies, longer reports, multiple reports, or projects with small teams		✓

## Data Availability

All available data are provided in the text or supplemental, no further data are available.

## Supplementary Materials

The following are available online at https://www.mdpi.com/article/10.3390/ijerph18116104/s1, MCR: open-source software to automate compilation of health study report-back.
