# Supplementary material for: MCR: Open-Source Software to Automate Compilation of Health Study Report-Back
Source: Int J Environ Res Public Health. 2021 Jun 5;18(11):6104. doi: 10.3390/ijerph18116104 (PMC8201126; doi:10.3390/ijerph18116104)
Supplement: Supplementary file 1 [file ijerph-18-06104-s001.zip › ijerph-1206873-supplementary.pdf]

MCR: open-source software to automate compilation of health study report-back

Erin Polka [a], Ellen Childs [b,c], Alexa Friedman [a], Kathryn S. Tomsho [d], Birgit Claus Henn [a], Madeleine K. Scammell [a], Chad W. Milando [a] *

* corresponding author: cmilando@bu.edu

Supplementary materials

Below is a report-back template and example "individualized" report (filled in with dummy data). The graphics on both pages were created separately, saved as images, and pasted into the template using the MCR. This figure demonstrates the capabilities of MCR to insert text and images into a report-back template (rather than serve as an example of report-back best practices).

Page 1:

Report-back template

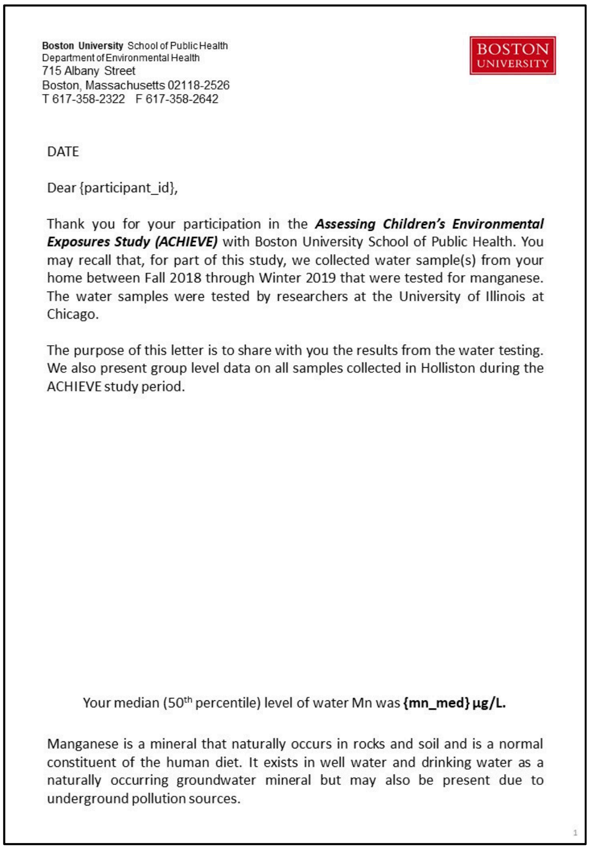

Boston University School of Public Health
Department of Environmental Health
715 Albany Street
Boston, Massachusetts 02118-2526
T 617-358-2322 F 617-358-2642

BOSTON UNIVERSITY

DATE

Dear {participant_id},

Thank you for your participation in the ***Assessing Children's Environmental Exposures Study (ACHIEVE)*** with Boston University School of Public Health. You may recall that, for part of this study, we collected water sample(s) from your home between Fall 2018 through Winter 2019 that were tested for manganese. The water samples were tested by researchers at the University of Illinois at Chicago.

The purpose of this letter is to share with you the results from the water testing. We also present group level data on all samples collected in Holliston during the ACHIEVE study period.

Your median (50th percentile) level of water Mn was **{mn_med} µg/L.**

Manganese is a mineral that naturally occurs in rocks and soil and is a normal constituent of the human diet. It exists in well water and drinking water as a naturally occurring groundwater mineral but may also be present due to underground pollution sources.

1

Compiled report

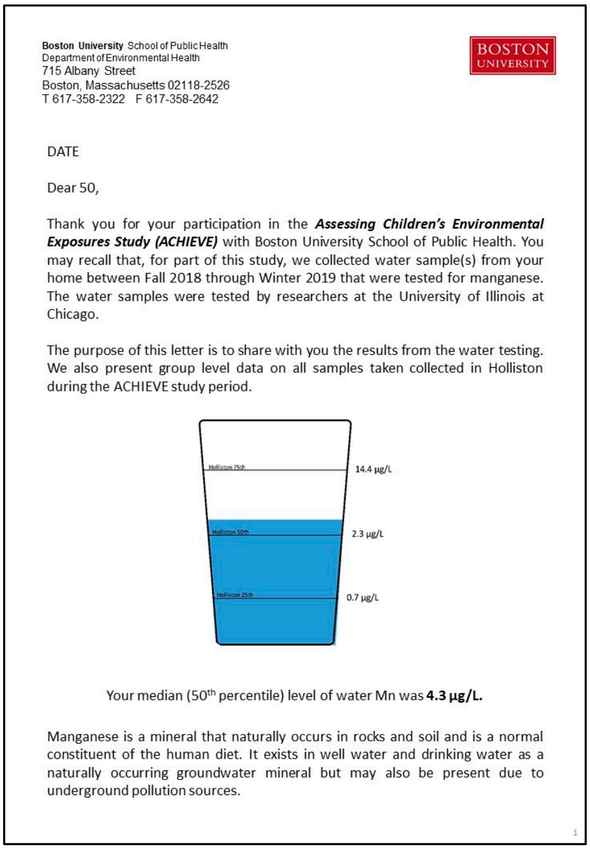

Boston University School of Public Health
Department of Environmental Health
715 Albany Street
Boston, Massachusetts 02118-2526
T 617-358-2322 F 617-358-2642

BOSTON UNIVERSITY

DATE

Dear 50,

Thank you for your participation in the ***Assessing Children's Environmental Exposures Study (ACHIEVE)*** with Boston University School of Public Health. You may recall that, for part of this study, we collected water sample(s) from your home between Fall 2018 through Winter 2019 that were tested for manganese. The water samples were tested by researchers at the University of Illinois at Chicago.

The purpose of this letter is to share with you the results from the water testing. We also present group level data on all samples taken collected in Holliston during the ACHIEVE study period.

Your median (50th percentile) level of water Mn was **4.3 µg/L.**

Manganese is a mineral that naturally occurs in rocks and soil and is a normal constituent of the human diet. It exists in well water and drinking water as a naturally occurring groundwater mineral but may also be present due to underground pollution sources.

1

Page 2

Report-back template

The level measured in your water sample ranged between **{mn_min} µg/L** and **{mn_max} µg/L**. {text_count} of the samples taken from your home were above the current EPA Secondary Drinking Water Maximum Contaminant Level (MCL) of 50 µg/L. Secondary Drinking Water MCLs are based on color, taste and odor. Manganese can change the color of your water (black or brown in color), cause staining, and cause the water to have a bitter metallic taste.

At this time, we do not know if there are any health risks associated with the amount of manganese in your water. If you are concerned about the amount of manganese in your water, please contact us for more information. Our contact information is listed below.

Again, we greatly appreciate your participation. We hope to continue working with you in the future!

Sincerely,

Department of Environmental Health
Boston University School of Public Health
ACHIEVE study email: **achievestudy2017@gmail.com**

2

Compiled report

Sample Date	Mn Level (µg/L)
September 25th, 2018	2.3
February 12th, 2019	**57.0**
March 21st, 2019	4.3
October 12th, 2019	**105.3**
October 29th, 2019	7.0
November 2nd, 2019	0.4
November 12th, 2019	5.60
December 1st, 2019	2.9
December 20, 2019	0.4

The level measured in your water sample ranged between **0.4 µg/L** and **105.3 µg/L.** Two of the samples taken from your home were above the current EPA Secondary Drinking Water Maximum Contaminant Level (MCL) of 50 µg/L. Secondary Drinking Water MCLs are based on color, taste and odor. Manganese can change the color of your water (black or brown in color), cause staining, and cause the water to have a bitter metallic taste.

At this time, we do not know if there are any health risks associated with the amount of manganese in your water. If you are concerned about the amount of manganese in your water, please contact us for more information. Our contact information is listed below.

Again, we greatly appreciate your participation. We hope to continue working with you in the future!

Sincerely,

Department of Environmental Health
Boston University School of Public Health
ACHIEVE study email: **achievestudy2017@gmail.com**

2
